# 2467. Automated Genomic Healthcare Epidemiology with One Codex

**DOI:** 10.1093/ofid/ofad500.2085

**Published:** 2023-11-27

**Authors:** Austin Davis-Richardson, Petras Zdanavicius, Timothy Reynolds, Terri Stillwell, Amanda Valyko, Laraine Washer, Jackie White, Shawn Hawken

**Affiliations:** One Codex, San Francisco, California; One Codex, San Francisco, California; One Codex, San Francisco, California; University of Michigan Health, Ann Arbor, Michigan; University of Michigan Health, Ann Arbor, Michigan; University of MIchigan, Ann Arbor, Michigan; University of Michigan Health, Ann Arbor, Michigan; One Codex, San Francisco, California

## Abstract

**Background:**

Genomic epidemiology is promising for precision Healthcare Associated Infection (HAI) tracking, and reducing the cost of infection prevention. Despite growing access to whole-genome sequencing (WGS), genomic healthcare epidemiology is not mainstream. For healthcare genomic epidemiology to become standard, tools for analysis and interpretation that are automated, reproducible, and accessible are required.

**Methods:**

We have extended One Codex to support analysis and interactive data visualization for genomic pathogen surveillance and outbreak investigation. First, these capabilities were evaluated for concordance with previous results including probable transmission, strain, antimicrobial resistance and virulence marker detection. Next, we describe the use of One Codex Genomic Epidemiology to investigate a potential HAI cluster of *Bacillus cereus* in a neonatal intensive care unit (NICU) at an academic medical center.

**Results:**

We developed a platform integrating sequencing, analysis, health record tracking, interactive visualization and interpretation **(Figure 1)**. WGS data can be generated or uploaded manually or through an interface with lab information systems. Data QC, taxonomic classification, antimicrobial resistance and strain detection, variant calling and phylogenetics are automatically conducted. Analysis modes include an epidemiologic curve, patient exposure and location history across isolate phylogenies (**Figure 2**). Clinical and sequence data and analysis results are versioned, allowing for review and audit, and are available through the application programming interface. Investigation of datasets demonstrated concordance with previous results (**Table 1**). Evaluation of a *B. cereus* cluster revealed unlikely transmission involving two patients and an environmental NICU isolate. This analysis pinpointed a single plausible transmission event requiring further investigation by Infection Prevention (**Figure 2)**.

**Figure:**
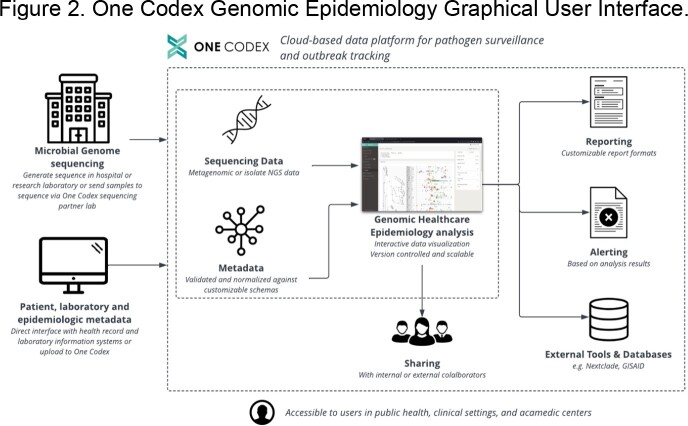

Investigation of a B. cereus cluster using the Genomic Epidemiology interface showing summary of SNP distances, spatiotemporal information for patients, MLST designation, epidemiologic curve and metadata summary. Patients 1 and 3 had isolates from MLST 73 which had a pairwise distance of 17 core SNPs, warranting further investigation by infection prevention to evaluate plausibility of transmission.

**Figure:**
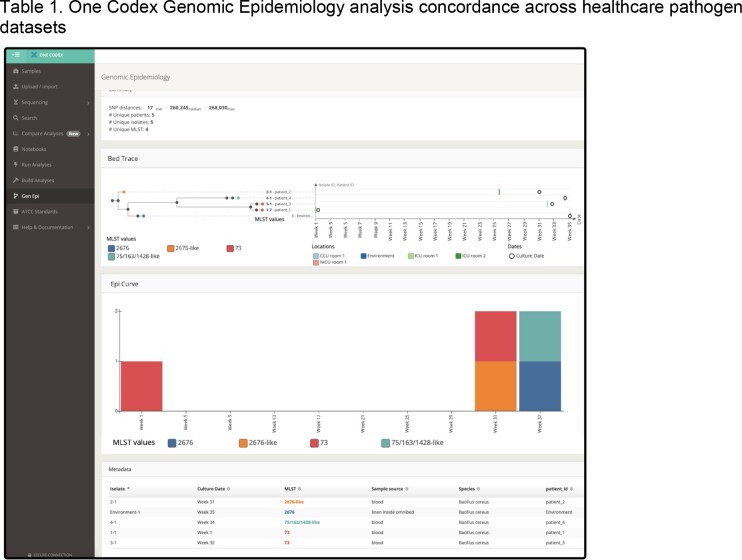

**Conclusion:**

One Codex facilitated accurate, and reproducible sequence to epidemiologic insight. Applications include evaluation of prospective surveillance, early outbreak detection, and genomics-driven deployment of infection prevention resources.

**Figure:**
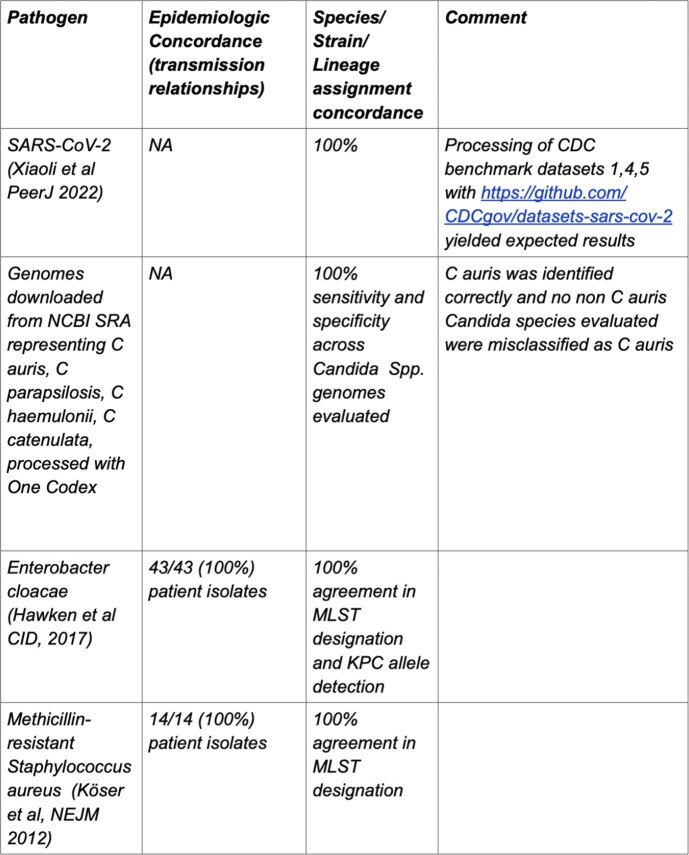

**Disclosures:**

**Austin Davis-Richardson, PhD**, Invitae: Employee|One Codex: employee **Petras Zdanavicius, MS**, Invitae: employee|One Codex: employee **Timothy Reynolds, PhD**, Invitae: Employee|One Codex: employee **Shawn Hawken, PhD, MPH**, Invitae: employee|One Codex: employee

